# Juvenile idiopathic arthritis and Wegener granulomatosis: causal or casual relationship?

**DOI:** 10.1186/1546-0096-9-S1-P175

**Published:** 2011-09-14

**Authors:** Balahan Makay, Tuba Tuncel, Nevin Uzuner, Erbil Ünsal

## 

The association of juvenile idiopathic arthritis (JIA) and Wegener granulomatosis (WG) is extremely rare. To date, only 3 patients with JIA accompanied by WG were described in the literature. Here we report a girl who had been followed-up for RF positive JIA for 3 years and developed WG at the age of 15. She started suffering from symmetrical arthritis of knees and wrists when she was 12. The tests for ANA, anti-ds DNA and ANCA were negative and RF was positive at that time. She was given methotrexate and oral prednisolon. Three years later, she complained of fatigue, weight loss and bloody sputum while she was taking methotrexate 15 mg/week. Her chest X-ray demonstrated cavitary lesions. A 10-day course of antibiotic therapy given by another center failed to improve her general condition. On admission to our hospital, her ESR was 110 mm/h and CRP was 193 mg/l. She had anemia, leukocytosis and thrombocytosis. Thorax CT revealed cavitary lesions on both lungs which may be consistent with opportunist infections. After obtaining bacterial, fungal and mycobacterial cultures of the sputum, she was started large spectrum antibiotics and antifungals. In the physical examination, she had saddle-nose. The biopsy of the nasal cavity revealed “necrotising granulomatous inflammation”. Her c-ANCA was positive. She was diagnosed as WG and started pulse prednisolon therapy and oral cyclophosphamide. After one-year follow-up, she is under remisson with oral azatiopurine and prednisolon.

